# Transforming a Patient Registry Into a Customized Data Set for the Advanced Statistical Analysis of Health Risk Factors and for Medication-Related Hospitalization Research: Retrospective Hospital Patient Registry Study

**DOI:** 10.2196/24205

**Published:** 2021-05-11

**Authors:** Zhivko Taushanov, Henk Verloo, Boris Wernli, Saviana Di Giovanni, Armin von Gunten, Filipa Pereira

**Affiliations:** 1 Faculty of Social and Political Sciences University of Lausanne Lausanne Switzerland; 2 Faculty of Psychology and Educational Sciences University of Geneva Geneva Switzerland; 3 School of Health Sciences HES-SO Valais-Wallis Sion Switzerland; 4 Service of Old Age Psychiatry Lausanne University Hospital Lausanne Switzerland; 5 FORS, Swiss Centre of Expertise in the Social Sciences University of Lausanne Lausanne Switzerland; 6 Pharmacy Benu Tavil-Chatton Morges Switzerland; 7 Institute of Biomedical Sciences Abel Salazar University of Porto Porto Portugal

**Keywords:** cluster analysis, hierarchical 2-step clustering, registry, raw data, hospital, retrospective, population based, multidimensional

## Abstract

**Background:**

Hospital patient registries provide substantial longitudinal data sets describing the clinical and medical health statuses of inpatients and their pharmacological prescriptions. Despite the multiple advantages of routinely collecting multidimensional longitudinal data, those data sets are rarely suitable for advanced statistical analysis and they require customization and synthesis.

**Objective:**

The aim of this study was to describe the methods used to transform and synthesize a raw, multidimensional, hospital patient registry data set into an exploitable database for the further investigation of risk profiles and predictive and survival health outcomes among polymorbid, polymedicated, older inpatients in relation to their medicine prescriptions at hospital discharge.

**Methods:**

A raw, multidimensional data set from a public hospital was extracted from the hospital registry in a CSV (.csv) file and imported into the R statistical package for cleaning, customization, and synthesis. Patients fulfilling the criteria for inclusion were home-dwelling, polymedicated, older adults with multiple chronic conditions aged ≥65 who became hospitalized. The patient data set covered 140 variables from 20,422 hospitalizations of polymedicated, home-dwelling older adults from 2015 to 2018. Each variable, according to type, was explored and computed to describe distributions, missing values, and associations. Different clustering methods, expert opinion, recoding, and missing-value techniques were used to customize and synthesize these multidimensional data sets.

**Results:**

Sociodemographic data showed no missing values. Average age, hospital length of stay, and frequency of hospitalization were computed. Discharge details were recoded and summarized. Clinical data were cleaned up and best practices for managing missing values were applied. Seven clusters of medical diagnoses, surgical interventions, somatic, cognitive, and medicines data were extracted using empirical and statistical best practices, with each presenting the health status of the patients included in it as accurately as possible. Medical, comorbidity, and drug data were recoded and summarized.

**Conclusions:**

A cleaner, better-structured data set was obtained, combining empirical and best-practice statistical approaches. The overall strategy delivered an exploitable, population-based database suitable for an advanced analysis of the descriptive, predictive, and survival statistics relating to polymedicated, home-dwelling older adults admitted as inpatients. More research is needed to develop best practices for customizing and synthesizing large, multidimensional, population-based registries.

**International Registered Report Identifier (IRRID):**

RR2-10.1136/bmjopen-2019-030030

## Introduction

The transition from paper-based patient records to electronic health records has provided unprecedented access to vast amounts of diverse clinical and health data at the point of care [1]. Undoubtedly, this transition offers a huge opportunity to exploit patient registries for scientific, clinical, and health-policy purposes. An electronic health record is the systematized collection of patients’ digitally stored health information. The term *patient registry* is generally used to distinguish registries focused on health information from other data sets, but there is currently no consistent definition in use [2]. The World Health Organization (WHO) describes registries in health information systems as “a file of documents containing uniform health information about individual persons, collected in a systematic and comprehensive way, in order to serve a predetermined purpose” [3]. Properly designed and executed patient registries can provide a real-world view of clinical practice, patient outcomes, safety, and comparative effectiveness [4,5]. Several national registries (eg, the National Committee on Vital and Health Statistics, or the Agency for Healthcare Research and Quality, both in the United States) are used for a broad range of purposes in public health and medicine as part of “an organized system for the collection, storage, retrieval, analysis, and dissemination of information on individual persons who have either a particular disease, a condition (eg, a risk factor) that predisposes the occurrence of a health-related event, or prior exposure to substances (or circumstances) known or suspected to cause adverse health effects” [1]. Other terms used to refer to patient registries are clinical registries, clinical data registries, disease registries, and outcomes registries [5,6]. A patient registry can be a powerful tool for observing the course of a disease, understanding variations in treatment and outcomes, examining factors that influence prognosis, describing care patterns, including the appropriateness of care and disparities in its delivery, assessing effectiveness, monitoring safety and harm, and measuring some aspects of the quality of care [1,6].

National and international statistics document elevated rates of hospitalization and emergency department admissions among polymedicated, home-dwelling older adults with multiple chronic conditions, and these are often caused by medication-related problems (MRPs) [7-10]. However, the determining factors of medication-related hospitalizations are poorly understood and require more investigations based on existing patient data [11]. The associations between age, comorbidities, polypharmacy, and adverse effects on health outcomes and health care consumption have been reported in multiple studies of emergency departments and hospitals, but the underlying mechanisms have often been unclear [12-14]. Several studies have demonstrated that one-quarter of the emergency department admissions for polymedicated, home-dwelling older adults are related to the inappropriate prescription of medicines or unsatisfactory medication management [15,16]. Poor medication management, inappropriate medicine prescription, and drug–drug interactions are frequent causes of admission [17,18]. The risk of MRPs increases not only with old age and comorbidities but also with the number of medications prescribed and with certain classes of medicines, such as medicines for cardiovascular diseases and diabetes [9,19]. The mechanisms behind those high rates of hospitalization in relation to MRPs deserve more attention. More knowledge and understanding of the factors predisposing and precipitating hospitalization and MRPs among polymedicated, home-dwelling older adults are needed too.

This paper aims to describe the method used to transform and synthesize a raw, multidimensional, patient registry data set to prepare it for exploitation as a database with which to examine predictive and survival analysis among hospitalized older inpatients.

## Methods

### Study Design

This multidimensional, retrospective, patient registry–based study explored the methods required to transform and synthesize a raw data set into a suitable database for further analysis of descriptive, predictive, and survival statistics to identify the risk factors that might induce MRPs among discharged, polymedicated older inpatients.

### Population and Sample

The multidimensional patient registry included 140 variables routinely collected during hospital stays by older adult inpatients aged 65 years old or more, living at home before hospitalization, with at least five prescribed medicines at discharge from hospital. The extracted data set was composed of a sample of 20,422 hospitalizations from 2015 to 2018, with similar numbers of annual hospitalizations: 5134, 5095, 5125, and 5068, respectively.

Medicines prescribed before hospital admission were not considered in the analysis due to a lack of data accuracy and validity. Indeed, information on medication at hospital admission is often collected from patients themselves, who may not accurately report their prescriptions, particularly in cases of unplanned hospitalization.

### Data Set Extraction and Importing

The hospital data set was extracted from a public teaching hospital’s registry, delivered to the investigators in a CSV (.csv) format file via an encrypted email and saved on a secure server. Finally, the data set was imported into the *R* statistical package for cleaning, data transformation, and synthesis [20]. Routinely collected data included information derived from patients’ medical and clinical statuses (patient-reported data, clinical examination, medical diagnoses, or medicines prescribed). The data set had to be cleaned up and synthesized to be suitable for analyzing descriptive, predictive, and survival statistics.

### Data Cleaning and Transformation

Clinical coding was carried out directly by health care professionals during routine daily care, using a pre-established drop-down menu. Official clinical coding of established medical (10th revision of the International Statistical Classification of Diseases and Related Health Problems [ICD-10]) and surgical diagnostics (CHOP) is mandatory under Swiss Federal Office of Public Health regulations. The variables represented by free text in the original database were excluded.

The distributions of each variable in the data set were explored, according to type (categorical and continuous variables), in order to identify any extreme values and obtain a better view of missing values and associations. Our data cleaning and transformation were guided by a literature review on cleaning-up large data sets, the quantity of information available to us, and the study aim [21]. One major challenge was to find a way to select or summarize a significant volume of information so that further descriptive and predictive statistical analyses could be performed (ie, summarize as many variables as possible, while losing the least amount of information). The large number of variables describing an inpatient’s somatic and cognitive status and medical diagnoses represents a significant challenge: we must find a balance between the variability of data and the essential, detailed information they provide without losing the ability to perform descriptive, predictive, and survival analyses [22].

### Presentation of the Data Set

#### Description of the Sociodemographic and Hospitalization Data Set

The sociodemographic data set—almost exclusively composed of ordinal variables—included just 2 categorical variables (sex and place of discharge) and 1 continuous variable (age). There were no missing sociodemographic variables except among the place-of-discharge data.

The hospitalization data set included 2 continuous variables (date of entry and discharge) and 1 categorical variable (the personal identification data number [PID]). These 3 variables enabled us to compute the length of stay (LOS) and the frequency of hospitalization and rehospitalization, respectively. Rehospitalization rates were important health status indicators in relation to drug prescriptions. Many polymedicated, home-dwelling older adults were hospitalized more than once during the 4-year study period. Almost one-third (n=3678) of older inpatients were rehospitalized 3 times or more; a small fraction was hospitalized more than 9 times. We found 18 polymedicated, home-dwelling older adults who were rehospitalized 17 times and considered them as outliers. Besides computing the average age and hospital LOS, no other interventions were necessary to clean up this section of the data set. Our analyses found an almost equal distribution of men and women, with an average age close to 79 (SD 7.7). Most older inpatients were discharged home after an average LOS of about 10 days (Multimedia Appendix 1).

#### Description of the Somatic Data Set

Nurses routinely collect clinical data during hospitalization using a drop-down menu, and the data set was composed of 18 categorical variables: 16 measured as ordinal variables (mobility, changing position, falls in the last year, exhaustion, upper- and lower-body care, upper- and lower-body [un]dressing, eating, drinking, micturition and defecation-related movements, hearing, vision, verbal expression, and pain intensity) and 2 measured as nominal variables (altered gait and chronic pain). Missing values in the data set were resolved by recoding them as “not available” (NA; Multimedia Appendix 2).

#### Description of the Cognitive Data Set

Inpatients’ cognitive status was measured at an ordinal level using 5 categorical variables. More than 72.60% (14,826/20,422) of adults showed no deterioration in their cognitive status (Multimedia Appendix 3).

#### Description of the Medical Diagnoses and Surgical Interventions Data Set

This data set of medical information was composed of patients’ principal medical diagnosis and 4 secondary medical diagnoses (active or passive comorbidities), based on the WHO’s ICD-10 adopted by Switzerland’s health care system [23]. This was completed with the patient’s principal surgical intervention and 4 additional surgical interventions, based on Switzerland’s surgical classification system (named CHOP) [24]. This data set showed no missing values (Multimedia Appendix 4).

The data set has no specific coding for MRPs (the corresponding ICD-10 is “Poisoning by drugs, medicaments and biological substances”) [25].

#### Description of the Prescribed Medicines Data Set

The hospital data set showed that discharged patients had been prescribed 2370 different medicines. This huge number of medicines and their heterogeneous therapeutic focus needed a structured classification built based on best practices (Multimedia Appendix 5). Based on expert opinion and a literature review on medicine classification systems, we chose the Anatomical Therapeutic Chemical (ATC) classification system’s 14 top-level codes to structure the set of prescribed medicines [25,26] (Multimedia Appendix 6).

### Synthesizing the Raw Data Set

Summarizing the data set was especially challenging because most of the variables documented different parts of inpatients’ overall health status, with all the diverse dimensions of their somatic and cognitive conditions. Special attention was given to the large data set of prescribed medicinal treatments. In many fields, the most common means of coping with such difficulties is the use of statistical clustering, a technique which combines all the available information (all variables) to reveal one or several underlying dimensions or health concepts.

In addition, the data set’s large number of variables and dimensions made it extremely complex to investigate the relationships and interactions between the different somatic and cognitive variables. The data set should allow the analysis of the risks of adverse health outcomes and their relationships with the medicines prescribed. For this reason, computing every variable in the same model may not be the optimal modeling choice if we consider the multidimensional aspect and dependency between those variables. This is especially true if these variables are significant (*P*<.01) for the discrimination and discovery of mechanisms leading to rehospitalization and a nonreturn home due to medical conditions and MRPs. In the absence of any scientific models, this study used an empirical approach.

### Data Clustering

#### Overview

Little research to date has explored specific combinations or clusters of clinical data and health status. Our study’s objective was to transform and synthesize valuable inpatient health information (health concepts such as mobility), rather than to reduce the dimensions of the data. It is, therefore, worth considering a larger number of principal components in the analysis to explain a larger part of the data variability. Almost all the studies which have examined specific comorbidities start from a specific disease rather than examining all the co-occurring clinical and medical conditions [27,28]. Nosology clusters groups of diseases, disorders, or syndromes with meaningful associations into a type of classification, so that diseases, for example, within a cluster, are very similar to one another, but are dissimilar to diseases in other clusters [29]. Among older inpatients, some associations are useful for identifying those at risk of in-hospital adverse clinical events and death in relation to those disease or health-syndrome clusters.

A large variety of clustering methods exist in the literature. However, the majority are focused on either continuous or nominal data alone. Only a limited number of techniques and strategies manage to incorporate both variable types into the same clusters [30].

#### Distance Measurement

This approach aims to create a measure of the distance between individuals or sequences that includes nominal and continuous variables. The Gower distance is the most widely used distance measure, and it can be used to calculate the distance between 2 entities whose shared attribute has a mixture of categorical and numerical values [31]. However, because it uses a range of continuous variables to determine the distance and assumes that nominal variables have a distance of either 0 or 1, the Gower distance may underestimate the impact of continuous variables because they are valued at 1 much less often than nominal variables are. Furthermore, weightings are selected arbitrarily. However, they define each data type’s contribution to the overall distance. As with all distance measures, the Gower distance should be used as an input for clustering methods, such as k-means.

#### K-Means Method

The k-means algorithm is mainly used for continuous variables [32]. Several other applications, such as the *R* statistical package KAMILA [33], integrate different types of variables. In this case, it uses the probabilities of a multinomial distribution for the discrete variables. The continuous variable distribution is estimated using univariate kernel densities [34]. The probabilities resulting from both distribution types are added together to obtain a measure of how close an observation is to the center of each cluster.

#### K-Medoids Method

The k-medoids method is a more robust version of k-means [35]. The difference is that in k-medoids real data points are selected as cluster centers, whereas in k-means the centers are the computed averages. The PAM function in the *R* statistical package KAMILA is a popular application of this approach [33,34].

#### Multiple Correspondence Analysis

The standard method for clustering factor variables is multiple correspondence analysis [36]. This model is implemented in the FactoMineR and PCAmixdata *R* packages. It splits all factors into multiple binary variables and applies a type of principal component analysis. The principal components obtained are then usually clustered using a k-means algorithm.

### Hierarchical Cluster Analysis

Our data analysis strategy applied a hierarchical cluster analysis, using the ClustOfVar R package [37,38]. As with any statistical analysis, results of a hierarchical cluster should not be accepted as they first appear, but should be taken as suggestions or questioned instead. When the final set of groups of variables was defined, a statistical model to cluster the individuals within each group was applied. This created one new variable for each group, indicating the type of characteristics the individual displayed in his/her health status assessment. For example, if we separate the individuals into 3 groups according to their cognitive status, we might obtain a variable indicating that a person belongs to a group with significant, minor, or no cognitive impairment. This type of aggregated variable was used in our final analysis of risk factors.

Our analysis explored several different clustering methods. However, the results displayed here most often used the following variable clustering procedure. First, a one-factor analysis model was typically used; second, the most important latent factors were selected. At this stage, it was essential to obtain accurate clustering rather than reduce the dimensionality, which takes place in the final cluster partition. Third, these factors were considered as variables and served as the input to a k-means clustering algorithm. Finally, the number of clusters was then selected using the Rousseeuw silhouette statistic, also with regard to the interpretability of the resulting partition [39].

### Two-Step Clustering Framework

In this approach, *n* and *p* denote the numbers of the patients and health conditions (indicators), respectively. The data can thus be represented by an *n *× *p* matrix, where the observed value for the *i*th column and the *j*th row of the data matrix is 1 or 0, indicating the presence or absence of the *i*th health condition for the *j*th respondent (*i *= 1,…, *p*; *j *= 1,…, *n*).

In the 2-step clustering approach, step 1 involves clustering the *p* conditions into non-overlapping groups of clinical or health conditions. Based on individual patterns in these groups of clinical and medical conditions, step 2 involves clustering the *n* respondents into clusters which correspond to different patterns of clinical or health conditions.

To thoroughly analyze the data and identify the MRPs leading to adverse health outcomes—such as rehospitalization, nonreturn home, and early death [40,41]—among older adult inpatients, a literature review was conducted [27].

### Treatment of Missing Data

As in every real-life data collection exercise, missing values are unavoidable, and it is important to define how these are integrated into the study. Four approaches were considered: ignoring all observations with 1 or more missing values; defining “NA” as a separate potential variable value; replacing every missing value by the mode of the corresponding variable; or performing multiple imputations on the data set. The first approach was obviously inappropriate, especially in cases where the number of missing data was significant (*P*<.01). Considering NA as a separate modality for each variable inflates the number of modalities, but it reduces the possibility of bias due to incorrect imputation methods. Nevertheless, for the sake of comparison, it was also tempting to consider the 2 latter approaches. Before choosing between simple replacement using the variable’s mode value and multiple imputation, we had to test for the type of missing data. If data are missing completely at random, we can simply impute using the mode. However, if this possibility is rejected, multiple imputation is theoretically more appropriate. The Little test (1988) [42] examines the null hypothesis H0: the data are missing completely at random. This test was applied to all subclusters of variables and the null hypothesis was rejected for every data set. This indicated that multiple imputation could be performed as an optional solution for estimating missing values.

Finally, defining NA values became our primary choice for the treatment of missing values. By creating an NA variable (an empty variable that does not influence the cluster result), all observations with an NA variable were still taken into account in the cluster analyses. This is why each cluster analysis contains every hospitalization (N=20,422).

### Ethical Considerations

The hospital data set was coded and its use was contractually limited by the participating hospital center. Furthermore, because the data sets included highly sensitive electronic patient records from a hospital registry, ethical approval was sought before any synthesis or analysis. Data were stored on a dedicated secure data server, which included a log registry. Each access flow to the secure data environment was documented, and each change required approval. Only users working on the project and requiring access to the data were allowed to use the selected multifactor authentication mechanism in the secure environment. The Human Research Ethics Committee of the Canton of Vaud (CER-VD) (2018–02196) approved the study on February 1, 2019.

## Results

### Transformation of the Data Set

The original data set required some adjustments before our plan of analysis could move forward. Four empty variables and 1 observation containing mostly 0 or unavailable values were removed from the data set. The labels for all variables were rewritten and clarified, and many medicine names in French had accents and unreadable symbols corrected.

### Missing Data

Tests made using both the BaylorEdPsych and RBtest *R* packages confirmed that the missing-completely-at-random hypothesis could be rejected [42]. Observations within each subcluster of the data set that only contained missing values were recoded as NA. Their presence might have been due to incorrect inputs, human or software error, or unavailable parts of some questionnaires. Missing data had very little impact on the sample size, appeared to be random, and concerned the first 4300 observations, especially. After recoding these observations, the cognitive status variables showed no more separate missing observations, and we had a complete data set.

### Clustering of Clinical and Medical Data

Most of the hospital variables were partially independent and gathered into several groups according to the dimension of the patient’s measured/assessed clinical and medical status. We used an empirical approach suggested by health care experts (FP, HV, and AvG) in an attempt to present homogenous groups within the set of variables. In cases involving clear and meaningful clustering, we relied on expert recommendations or opinions taken from a comprehensive literature review [27,33]. However, when evidence was scarce, we clustered variables using statistical methods. The results from statistical methods were compared against those from expert opinion, which served as a validation tool for addressing any possible subjectivity in those expert opinions [27,33].

**Figure 1:**
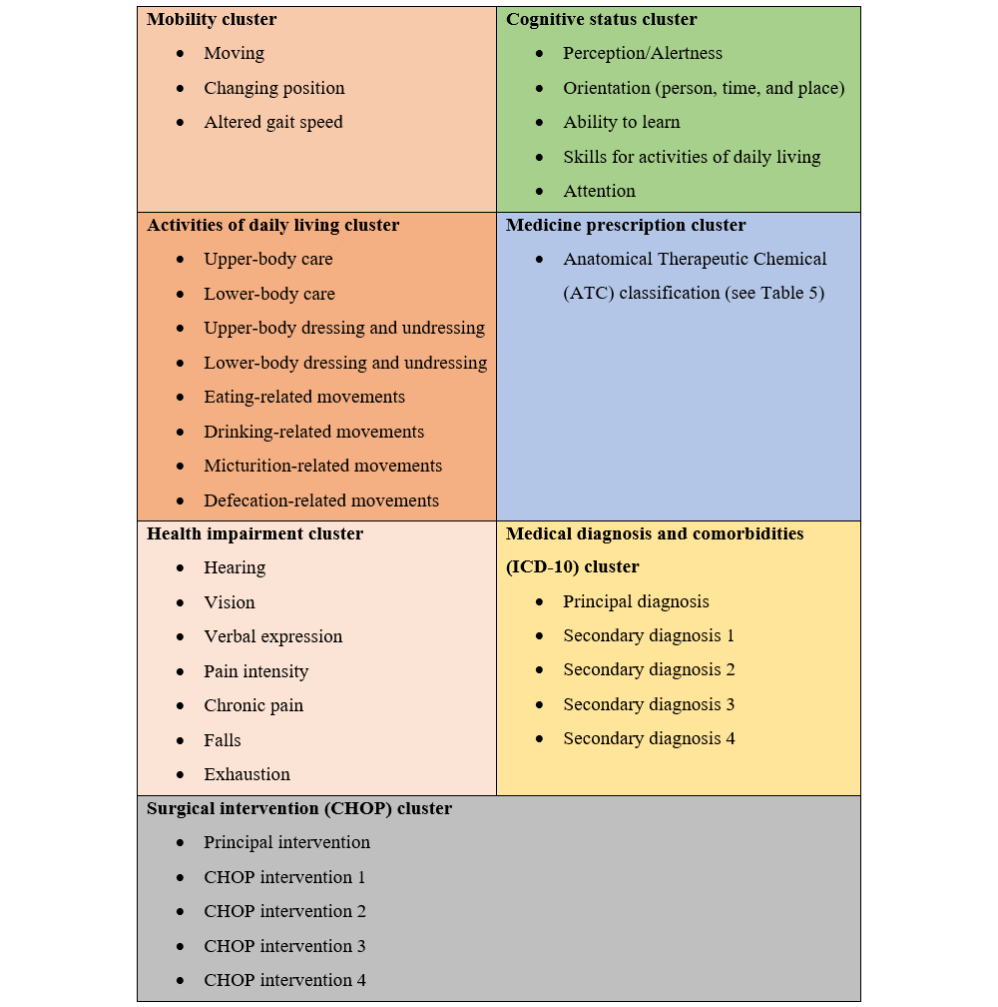
Structure and content of the data set clusters.

Seven groups of clusters were developed: somatic/physical health conditions (3 orange groups in Figure 1), cognitive health conditions (green textbox in Figure 1), total number of prescribed medications based on the ATC classification, diagnoses based on the ICD-10 (yellow textbox in Figure 1), and the surgical interventions based on CHOP (gray textbox in Figure 1). Besides these more apparent distinctions between variables, other underlying subclusters may be present within these groups. This point is beyond the scope of this paper, however, and will be documented elsewhere with a complementary, within-group analysis (the presence of an interpretable clustering of variables within a group before clustering individuals). An examination of the *place of discharge* variable confirms this: of 20,422 hospitalizations, only 131 patients (<1%) were documented to have died during hospitalization. Bearing in mind that there was no explicit variable indicating this worst outcome, we developed indicators that were suggestive of imminent death or a highly and irreversibly deteriorated health condition. Based on a literature review of polymorbidity, 6 clinical indicators from the data set were associated with a functional deterioration leading to progressive decline and poor health status [43]: (1) restricted mobility, (2) incapacity to change position, (3) altered alertness, (4) altered orientation, (5) altered gait, and (6) reduced or absent cognitive skills necessary to carry out the activities of daily living. Each of these variables indicated a deteriorating health status. To ensure that only severely deteriorating health problems were captured, we only considered patients to be endangered if they had multiple problems. We therefore created a variable indicating the number of problems present, with values ranging from 0 to 6 (Multimedia Appendix 7). More than half of the sample presented with at least one deteriorated health condition. However, only a small fraction of the older adult patients had 4 or more deteriorated health conditions at discharge.

### Cognitive Data Cluster

#### Overview

The cognitive data cluster (green textbox in Figure 1) was composed of 5 variables indicating cognitive status level (Table 1). As with many other variables in the total data set, cognitive data were considered nominal because they each had a small number of modalities. The first 400 observations in the data set were excluded from the cognitive status analysis because they contained only missing values and were excluded from other analyses for the same reason. These missing values were explained by the fact that new data variables were introduced into the hospital register during the first semester of 2015.

#### Cognitive Status Clustering

**Figure 2:**
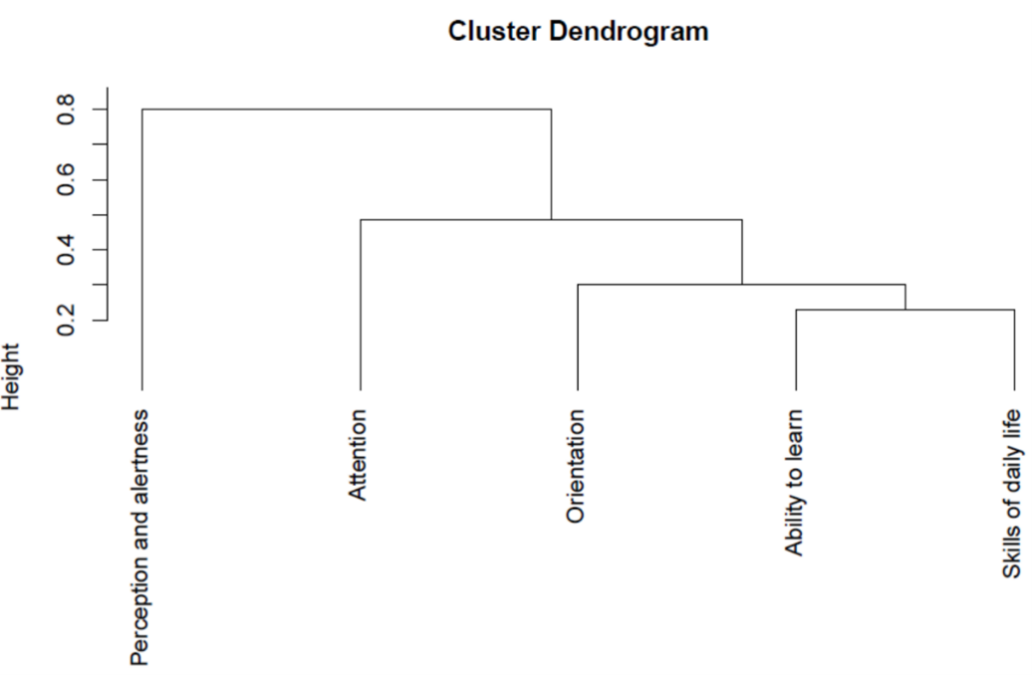
Dendrogram of cognitive status variables.

The *R* ClustOfVar package was used to perform a hierarchical clustering of the cognitive health variables to investigate any possible relationships and the presence of subclusters within these variables. The results did not suggest any clear interpretable structure within the variables included, as illustrated by the dendrogram (Figure 2). They indicated that only single-variable clusters (singletons) could be separated, one at a time, to form separate and not very distinct clusters. This information failed to provide any useful solution to our problem because it makes no sense to cluster individuals using a single variable. This result, combined with the small total number of 5 other data set clusters, led us to the conclusion that the 6 data set clusters illustrating different cognitive conditions should be considered together in the same clustering algorithm.

Multiple correspondence analysis was used to cluster individuals according to their cognitive status because all the variables were categorical. Even though the first 2 principal components do not explain much of the data (5310/20,422, 26.00%), we were able to discern the 4 most discriminant variables for clustering (and the importance of their categories). For further analysis, we selected numerous principal components (n=9) because of their relatively low explanatory power (65% of the variance). We found multiple different clustering partitions with respect to the number of clusters. Some groups and features were found systematically in all the partitions. This enabled us to make the following generalizations about the results, regardless of the number of clusters:

- The majority of observations indicated that cognitive status was not altered at the time of the assessment. We found a good solution and form in every cluster, including the largest cluster.
- When increasing the number of clusters, observations with average or poor cognitive status were split and nuanced.
- One group of individuals with mainly missing values was excluded from the analysis.

**Figure 3:**
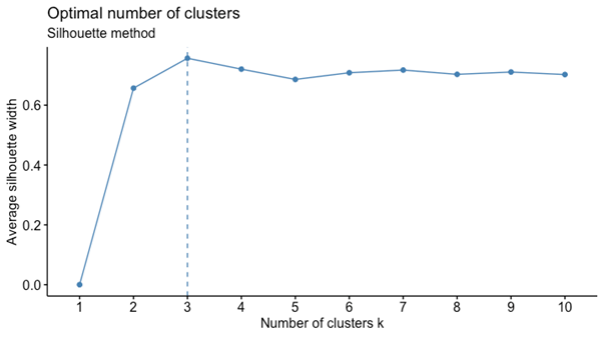
Silhouette statistics for choosing the optimal number of clusters: the two- or four-cluster solutions were suggested.

The optimal number of clusters was determined using the silhouette statistic (Figure 3). For each number of clusters, this statistic measures how similar each observation is to its own cluster in comparison to all other clusters, that is, the extent to which observations are grouped together. The results indicated that the 3-cluster solution would be the most appropriate in terms of within- and between-cluster distances. However, a partition using 2 clusters provided greater simplicity and also had a statistically sustainable silhouette value.

#### Two-Cluster Solution

Hierarchical clustering using 2 classes created a dominant group of 18,339/20,422 (89.80%) older inpatients with full cognitive ability and a smaller group of 2083/20,422 (10.20%) inpatients with cognitive impairment. The 2-cluster solution was differently distributed over the 5 variables and according to the type of diagnoses (ICD-10; Table 1), and it was highly significant (*P<*.001). Two other variables (number of medications prescribed and primary diagnosis) were added to the analysis for experimental purposes but were not included in the clustering model. A difference was observed in the average number of medications prescribed (9.63 vs 10.47; *P*<.001) between groups, and the primary diagnosis also appeared to be different (0.10 vs 0.08; *P*<.001; Table 1).

**Table 1:** Distribution of individuals in each group for all 5 cognitive status variables in the 2-cluster solution (N=20,422).

Cognitive status variables			Cognitive status	
			Full ability	Cognitive impairment
**Perception/Alertness ^a^**				
	Alert	1.00		0.85
	Drowsy	0.00		0.13
	Stupor	0.00		0.01
	Coma	0.00		0.01
	NA^b^	—		—
	Distribution, n (%)	18,318 (89.70)		2083 (10.20)
**Orientation^a^**				
	Full ability	0.91		0.11
	3 abilities	0.08		0.24
	1–2 abilities	0.01		0.40
	Inability	0.00		0.20
	NA	0.00		0.06
	Distribution, n (%)	18,319 (89.70)		2083 (10.20)
**Ability to learn^a^**				
	Full ability	0.81		0.02
	Slightly reduced	0.18		0.10
	Severely reduced	0.02		0.67
	Inability	0.00		0.21
	NA	—		—
	Distribution, n (%)	18,319 (89.70)		2083 (10.20)
**Activities of daily living^a^**				
	Full ability	0.83		0.03
	Slightly reduced	0.15		0.16
	Severely reduced	0.02		0.66
	Inability	0.00		0.13
	NA	0.00		0.01
	Distribution, n (%)	18,319 (89.70)		2083 (10.20)
**Attention**				
	Unaffected	0.98		0.36
	Reduced	0.02		0.63
	NA	0.00		0.01
	Distribution, n (%)	18,319 (89.70)		2083 (10.20)
**Number of medicines^a^**				
	Average number	9.63		10.47
**ICD-10^c^ main diagnoses^a^**				
	Systems	0.52		0.54
	Mental	0.10		0.08
	Cancers	0.01		0.01
	Other	0.37		0.37
	NA	—		—
	Distribution, n (%)	18,339 (89.80)		2083 (10.20)

^a^Variables significantly different among clusters (χ^2^ tests and *t* tests, *P*<.01). Each line represents 1 cluster and adds up to 1 (100%).

^b^NA: not available.

^c^ICD-10: 10th revision of the International Statistical Classification of Diseases and Related Health Problems.

#### Three-Cluster Solution

Hierarchical clustering using 3 classes created groups of 15,717/20,422 (76.96%) polymedicated older inpatients in full cognitive health, 4290/20,422 (21.01%) with mild cognitive impairment, and 415/20,422 (2.03%) with severe cognitive impairment. The 3-cluster solution’s results were similar to those of the 2-cluster solution (Table 2).

**Table 2:** Distribution of individuals in each group for all 5 cognitive status variables in the 3-cluster solution (N=20,422).

Cognitive status variables		Cognitive status		
		Full ability	Mild cognitive impairment	Severe cognitive impairment
**Perception/Alertness^a^**				
	Alert	1.00	0.93	0.61
	Drowsy	0.00	0.07	0.29
	Stupor	0.00	0.07	0.06
	Coma	0.00	0	0.04
	NA^b^	—	—	—
	Distribution, n (%)	17,855 (87.43)	2166 (10.61)	380 (1.86)
**Orientation^a^**				
	Full ability	0.94	0.10	0.03
	3 abilities	0.06	0.39	0.05
	1–2 abilities	0.00	0.41	0.12
	Inability	0.00	0.08	0.62
	NA	0.00	0.02	0.18
	Distribution, n (%)	17,856 (87.44)	2166 (10.61)	380 (1.86)
**Ability to learn^a^**				
	Full ability	0.83	0.03	0.01
	Slightly reduced	0.17	0.23	0.03
	Severely reduced	0.01	0.70	0.09
	Inability	0.00	0.05	0.87
	NA			
	Distribution, n (%)	17,856 (87.44)	2166 (10.61)	380 (1.86)
**Activities of daily living^a^**				
	Full ability	0.85	0.06	0.01
	Slightly reduced	0.13	0.29	0.02
	Severely reduced	0.02	0.63	0.32
	Inability	0.00	0.02	0.62
	NA	0.00	0.00	0.03
	Distribution, n (%)	17,856 (87.44)	2166 (10.61)	380 (1.86)
**Attention^a^**				
	Unaffected	0.99	0.49	0.11
	Reduced	0.01	0.51	0.84
	NA	0.00	0.00	0.04
	Distribution, n (%)	17,856 (87.44)	2166 (10.61)	380 (1.86)
**Number of medicines^a^**				
	Average number	9.62	10.43	10.35
**ICD-10^c^ main diagnoses^a^**				
	Systems	0.52	0.54	0.57
	Mental	0.10	0.07	0.09
	Cancers	0.01	0.01	0.00
	Other	0.37	0.38	0.33
	NA	—	—	—
	Distribution, n (%)	17,876 (87.53)	2166 (10.61)	380 (1.86)

^a^Variables significantly different among clusters (χ^2^ tests and *t* tests, *P*<.01). Each line represents 1 cluster and adds up to 1 (100%).

^b^NA: not available.

^c^ICD-10: 10th revision of the International Statistical Classification of Diseases and Related Health Problems.

### Somatic Variables and Their Clustering Into Subclusters

Multiple variables showed modalities that did not correspond exactly to those described in the list (Multimedia Appendices 1-6). The *risk of falling* variable in the list of somatic data (orange textbox, Figure 1) is continuous, and it was thus recoded into a 3-modality factor as no risk (0 falls), moderate risk (1-4 falls), and high risk (≥5 falls in the last year).

The number of somatic variables is large and heterogeneous, making the direct clustering of individuals challenging. We considered the hypothesis that there were probably dissimilarities in this whole set of somatic variables, and starting from this assumption, we split the variables into subclusters.

**Figure 4:**
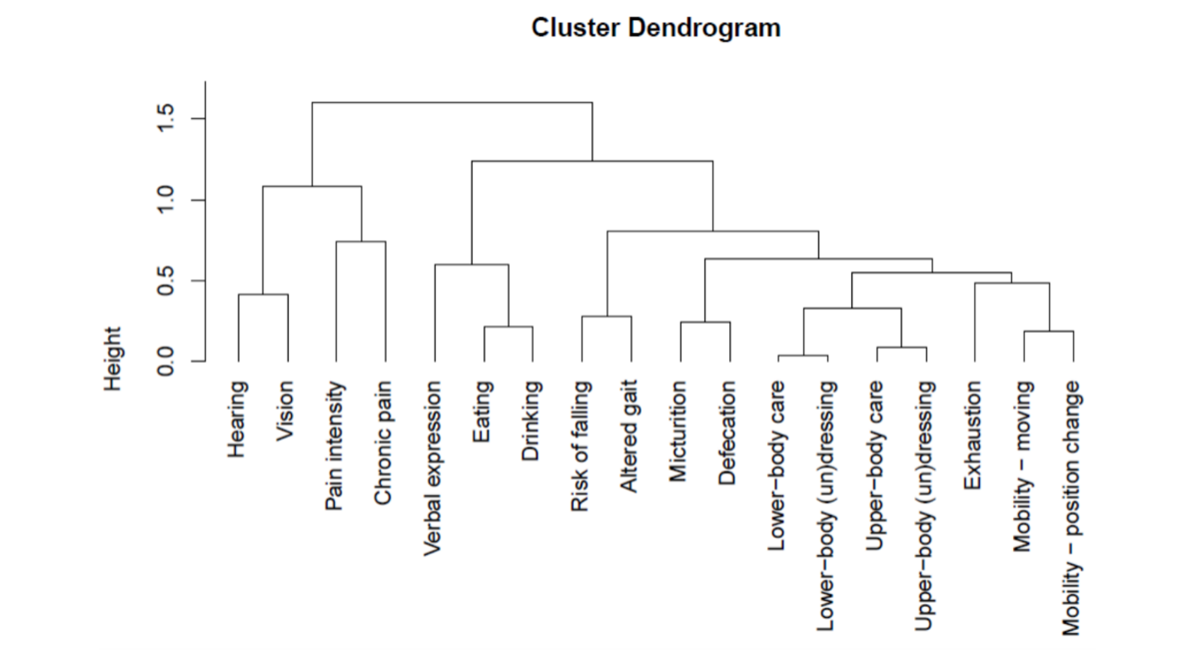
Dendrogram of the somatic health status variables.

In the absence of any validated techniques, tools, or evidenced-based literature, we developed an empirical subcluster clustering strategy. The initial separation of the variables was guided by information retrieved from a literature review of communicable somatic diseases completed with the authors’ experiences and expertise in patterns of somatic illness [27,28]. Four subclusters of somatic variables were constructed: mobility, health difficulties, capacities for the activities of daily living, and other health risks (orange textbox in Figure 1). The mobility subcluster was composed of the clinical variables of movement, changing position, altered gait, balance disorders, and past and recent falls. The general health status subcluster included exhaustion, hearing, vision, verbal expression, drowsiness, sleep rhythm, sleep impairment, pain intensity, and chronic pain. The capacities for the activities of daily living subcluster were composed of upper- and lower-body care, upper- and lower-body (un)dressing, eating, drinking, and micturition- and defecation-related movements. The other health risks subcluster was composed of clinical variables assessing the risks of sores, wounds, malnutrition, and falling during hospitalization. To reinforce the authors’ opinions, a statistical validation model of the variable clustering was computed using the hierarchical clustering functions of the *R* ClustOfVar package (Figure 4).

Findings showed some differences between the authors’ opinions and the statistical model. To optimize the composition of somatic health status variable subclusters, an adapted version was selected for further data analysis following discussion and a consensus agreement. Three subclusters of somatic variables were considered. The mobility subcluster was composed of the movement, changing position, and altered gait variables. The general health impairments subcluster included exhaustion, hearing, vision, verbal expression, risk of falling, chronic pain, and pain intensity. The capacities for the activities of daily living subcluster included upper- and lower-body care, upper- and lower-body (un)dressing, eating, drinking, and micturition- and defecation-related movements.

### Grouping Individuals Within the Somatic Health Status Subcluster

After separating the variables, the somatic health status subclusters of mobility, health impairments, and capacities for the activities of daily living were themselves partitioned, with the aim of discovering any possible underlying groupings of inpatients.

#### Mobility Subcluster

**Figure 5:**
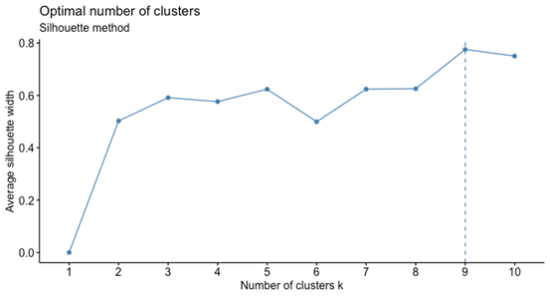
Average silhouette width for each number of sub-clusters in the mobility sub-cluster.

Using the silhouette statistic failed to give a clear optimal number of subgroupings n (Figure 5).

Our analysis demonstrated similar and increasing average silhouette widths as n increased. Consequently, we chose a 2-cluster partition, deciding that this best separated the variables in terms of interpretability of results and a clear implicit difference between the groups: a grouping of persons with mostly full mobility (n=12,540) and a grouping with an impaired mobility status (n=7,880). Roughly two-thirds of individuals had few or no mobility problems (Table 3). The remaining individuals exhibited problems in at least one of the three variables. That number is large but not surprising when considering the sample population’s advanced age. The χ^2^ tests confirmed a clear difference between the groups across all variables (Table 3). Our analysis highlighted that the group with full mobility status was prescribed significantly fewer medications (*P<*.01) than the group with impaired mobility (9.07 vs 10.74).

**Table 3:** Distribution of individuals in the 2-cluster solution for all mobility variables (N=20,422).

Mobility variables			Mobility status		
			Full mobility		Poor mobility
**Movement^a^**					
	Full ability	0.90		0.01	
	Slightly reduced	0.09		0.61	
	Severely reduced	0.00		0.30	
	Inability	0.00		0.08	
	Distribution, n (%)	12,540 (61.40)		7878 (38.58)	
**Changing position^a^**					
	Full ability	0.99		0.25	
	Slightly reduced	0.01		0.51	
	Severely reduced	0.00		0.21	
	Inability	0.00		0.04	
	Distribution, n (%)	12,540 (61.40)		7878 (38.58)	
**Altered gait speed^a^**					
	No	0.85		0.13	
	Yes	0.15		0.82	
	Not available	0.00		0.06	
	Distribution, n (%)	12,540 (61.40)		7878 (38.58)	
**Number of medicines^a^**					
	Average number	9.07		10.74	

^a^Variables significantly different among clusters (χ^2^ tests and *t* tests, *P<*.01). Each line represents 1 cluster and adds up to 1 (100%).

#### Health Impairments Subclusters

**Figure 6:**
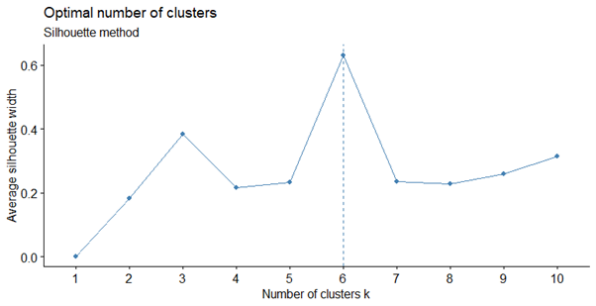
Health impairments sub-cluster: silhouette statistics for choosing the number of groupings suggested the four-cluster grouping solution.

Calculating the silhouette statistic suggested that the 4-cluster groupings solution was optimal, even though the results appear very surprising. However, we decided on the 2-grouping solution, mainly because it is easier to interpret (Figure 6 and Table 4).

**Table 4:** Distribution of individuals in the 2-cluster solution for all health impairment variables (N=20,422).

Health impairment variables		Health status	
		Good health status	Impaired health status
**Hearing^a^**			
	Full ability	0.88	0.77
	Auditive problems	0.12	0.22
	Deaf	0.00	0.10
	Distribution, n (%)	17,897 (87.64)	2465 (12.07)
**Vision^a^**			
	Full ability	0.92	0.73
	View problems	0.08	0.27
	Blind	0.00	0.01
	Distribution, n (%)	17,897 (87.64)	2465 (12.07)
**Verbal expression^a^**			
	Full ability	1.00	0.49
	Limited capacity	0.00	0.47
	Incapacity	0.00	0.04
	Distribution, n (%)	17,898 (87.64)	2465 (12.07)
**Risk of falling^a^**			
	No risk	0.37	0.05
	Moderate risk	0.63	0.34
	High risk	0.00	0.61
	Distribution, n (%)	17,844 (87.38)	2464 (12.07)
**Chronic pain^a^**			
	No pain	0.90	0.84
	Pain	0.10	0.15
	Not measurable	0.00	0.01
	Distribution, n (%)	17,872 (87.51)	2462 (12.06)
**Pain intensity^a^**			
	No pain	0.08	0.13
	Improbable	0.26	0.29
	Low	0.01	0.01
	Moderate	0.00	0.01
	Intense	0.00	0.01
	Pain index	0.65	0.55
	Distribution, n (%)	17,880 (87.55)	2462 (12.06)

^a^Variables significantly different among clusters (χ^2^ tests and *t* tests, *P*<.01). Each line represents 1 cluster and adds up to 1 (100%).

#### Capacities for the Activities of Daily Living Subcluster

**Figure 7:**
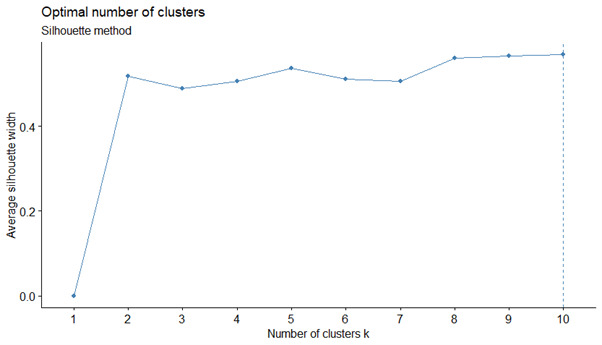
Silhouette statistics for the sub-cluster of capacities for the activities of daily living.

The 2-cluster solution appeared appropriate and confirmed the silhouette statistic, which highlighted the 2, 8, and 10-cluster solutions (Figure 7). We distinguished 1 large cluster grouping of 17,836/20,422 (87.34%) individuals composed of mainly *autonomous* inpatients with almost full capacity to carry out the majority of the activities of daily living. The second cluster grouping of more *dependent* inpatients included 2573/20,422 (12.60%) individuals with at least one serious problem in handling their activities of daily living. Overall, the partitioning into 2 cluster groupings was relevant in light of our aim to demonstrate that the observations were significantly different (*P*<.01) among the overall variables and in relation to the number of prescribed medications (Table 5).

**Table 5:** Distribution of the capacities for the activities of daily living subcluster (N=20,422).

Activities of daily living			Subclusters		
			Autonomous grouping		Dependent grouping
**Upper-body care^a^**					
	Full capacity	0.77		0.03	
	Slightly reduced	0.21		0.24	
	Severely reduced	0.02		0.47	
	Incapacity	0.00		0.26	
	Distribution, n (%)	17,836 (87.34)		2573 (12.60)	
**Lower-body care^a^**					
	Full capacity	0.61		0.00	
	Slightly reduced	0.25		0.01	
	Severely reduced	0.12		0.18	
	Incapacity	0.01		0.81	
	Distribution, n (%)	17,836 (87.34)		2573 (12.60)	
**Upper-body (un)dressing^a^**					
	Full capacity	0.80		0.01	
	Slightly reduced	0.18		0.16	
	Severely reduced	0.02		0.44	
	Incapacity	0.00		0.39	
	Distribution, n (%)	17,836 (87.34)		2573 (12.60)	
**Lower-body (un)dressing^a^**					
	Full capacity	0.64		0.00	
	Slightly reduced	0.22		0.01	
	Severely reduced	0.12		0.17	
	Incapacity	0.02		0.82	
	Distribution, n (%)	17,836 (87.34)		2573 (12.60)	
**Eating-related movements^a^**					
	Full capacity	0.95		0.35	
	Slightly reduced	0.05		0.38	
	Severely reduced	0.00		0.15	
	Incapacity	0.00		0.12	
	Distribution, n (%)	17,836 (87.34)		2573 (12.60)	
**Drinking-related movements^a^**					
	Full capacity	0.97		0.56	
	Slightly reduced	0.02		0.25	
	Severely reduced	0.00		0.12	
	Incapacity	0.00		0.08	
	Distribution, n (%)	17,836 (87.34)		2573 (12.60)	
**Micturition-related movements^a^**					
	Full capacity	0.85		0.12	
	Slightly reduced	0.11		0.19	
	Severely reduced	0.01		0.27	
	Incapacity	0.02		0.42	
	Distribution, n (%)	17,836 (87.34)		2573 (12.60)	
**Defecation-related movements^a^**					
	Full capacity	0.88		0.18	
	Slightly reduced	0.10		0.19	
	Severely reduced	0.02		0.33	
	Incapacity	0.01		0.3	
	Distribution, n (%)	17,836 (87.34)		2573 (12.60)	
**Number of medicines^a^**					
	Average number	9.48		11.39	

^a^Variables significantly different among clusters (χ^2^ tests and *t* tests, *P<*.01). Each line represents 1 cluster and adds up to 1 (100%).

### Synthesizing ICD-10 and CHOP Diagnoses

Clustering the large data set with more than 2000 different ICD-10 and 800 different CHOP diagnoses into general clusters was not interpretable. To make it suitable for further analysis, the ICD-10 data set was recoded into 4 groups: physiological systems, mental illnesses, oncological diseases, and others. The CHOP diagnoses were also recoded into 4 groups: physiological systems, sensorial, other, and measurement instruments for diagnostics (Table 6).

**Table 6:** Distribution of the recoded data set using the ICD-10 and CHOP diagnoses (N=20,422).

Diagnosis data set			Recoded data set										
			First		Second		Third		Fourth		Fifth		Total
**ICD-10^a^ diagnoses**													
	Physiological systems	10,666		10,311		10,277		10,034		9,495		50,783	
	Mental illnesses	2041		1181		856		609		465		5152	
	Oncological diseases	221		770		974		1012		1075		4052	
	Others	7490		7829		7308		6609		5768		35,004	
	No diagnosis	—		331		1008		2158		3619		7116	
	Total	20,418		20,422		19,415		20,422		20,422			
**CHOP diagnostics**													
	Physiological systems	5086		3656		2255		2049		1293		14,339	
	Sensorial	526		1448		1370		740		489		4573	
	Other	8535		4964		3222		1964		1503		20,188	
	Measurement instruments	—		23		22		1		—		46	
	Total	14,147		10,091		6869		4754		3285			
	No diagnosis/surgery	6275		10,331		13,553		15,668		17,137			

^a^ICD-10: 10th revision of the International Statistical Classification of Diseases and Related Health Problems.

### Summary of Synthesized Registry Data

The different clustering and recoding methods resulted in the data set presented in Table 7.

**Table 7:** Summary of the variables and clusters in the synthesized data set ready for further advanced statistical analysis.

Domain		Variables per cluster in the synthesized database	Recoding^a^ cluster level^b^	Inpatients in each cluster, n (%)
Sociodemographic characteristics (N=20,422)		6	—	20,422 (100.00)
Cognitive status (green textbox in Figure 1; n= 20,401)		5	2^b^	18,318 (89.79) and 2083 (10.21)
**Somatic status (orange textbox in Figure 1)**				
	Mobility subcluster (n=20,418)	3	2^b^	12,540 (61.42) and 7878 (38.58)
	Health impairments subcluster (n=20,362)	5	2^b^	17,897 (87.89) and 2465 (12.11)
	Activities of daily living subcluster (n=20,409)	5	2^b^	17,836 (87.39) and 2573 (12.61)
Medical condition ICD-10^c^ and CHOP (gray and yellow textboxes in Figure 1; N=20,422)		2,800	4^a^	Not applicable
Medicines (blue textbox in Figure 1; N=20,422)		2,370	14^a^	Not applicable

^a^Coded data.

^b^Clustered data (ability/impairment).

^c^ICD-10: 10th revision of the International Statistical Classification of Diseases and Related Health Problems.

## Discussion

### Principal Findings

This paper describes the rationale and methods used to synthesize a large, routinely collected data set of clinical and medical information concerning polymedicated home-dwelling older adults during hospitalization. The electronic patient records from a hospital center provided a valuable data resource for researchers wishing to perform a variety of analyses to explore health risk determinants, medication prescribing, rehospitalization, and death rates. Prospectively collecting research data is often time-consuming and expensive, resulting in biased samples of highly selected individuals, who are often unrepresentative of real-life patients [21]. Data that are already available for use in anonymized electronic patient records provide a valuable opportunity for a variety of different research designs and are particularly useful in the design of registries for evaluating patient outcomes [44]. In some situations, using population-based registries is even preferable to collecting primary data because selection bias due to nonresponders is not a problem [21]. However, large patient registries are sometimes also inconvenient as they frequently present raw data sets and, for several different reasons, they may not be immediately suitable for performing advanced statistical analyses [22]. Those large data sets usually need to be transformed, cleaned-up, and synthesized to be usable for advanced descriptive and predictive statistical analyses.

Our 4-year population-based data set was composed of polymedicated home-dwelling older inpatients with multiple chronic conditions, hospitalized and perhaps rehospitalized in a hospital center in the French-speaking part of Switzerland. The data came from multiple data set sources and were not easily exploitable for advanced statistical analyses, forcing the research team to explore and develop a synthesizing strategy for a large set of variables so as to respond to our research question. Synthesizing a large number of heterogeneous variables in a finite set of specific medical, clinical, and medication data groups was carried out using the principles of cluster methodologies [30,32] and following Olsen’s recommendations for best practices in the analysis of population-based registries [22]. Most of the variables documenting patients’ health status fulfilled the criteria for clustering into different groups according to the dimensions of their health status. Despite the existence of a large number of clustering algorithms, we observed that clustering variables remains a challenge [37]. First, our data set covered a large number of different domains, and it is often the case that clustering algorithms must be applied to heterogeneous sets of variables, creating an acute need for robust, scalable clustering methods for mixed continuous and categorical-scale data [45]. Current clustering methods for mixed-type data are generally unable to equitably balance the contributions of continuous and categorical variables without strong parametric assumptions. Second, stable cluster analysis is strongly dependent on the data set, especially on how well separated and how homogeneous the clusters are. In the same clustering exercise, some clusters will be more or less stable than others [46]. To overcome this challenge, our study used a combined empirical and statistical approach. In the empirical approach, the variables in the clusters and subclusters were selected following expert opinion (FP, HV, and AvG), presenting the most homogeneous groups possible within the set of variables described in the literature [47]. In the statistical approach, we used the most appropriate clustering methods and compared the results with the experts’ opinions, which served as a validation tool to address any possible subjectivity in those opinions. Both methods were implemented independently and compared. This approach was similar to that used in 2 recent studies exploring frailty and comorbidity patterns [27,28]. Although this study developed 6 clusters based on best practices and the previously mentioned empirical statistical approach, other underlying subclusters could also be present within them. This was also noted in the study by Newcomer et al [48] which used agglomerative hierarchical clustering methods to identify clinically relevant subclusters based on groupings of coexisting conditions in a large sample of hospitalized adults.

This study demonstrated that constructing subclusters should not rely solely on an explicit statement indicating the worst outcome, such as death. Clinical indicators documenting functional deterioration which led to a progressive decline and a poor health status were integrated into the 7 clustered data sets. A recent population-based registry study by Vuik et al [49] confirmed the utility of this kind of approach and concluded that health status could not only be based on sociodemographic characteristics and medical diagnoses such as age or morbidity, but should also consider specific assessments of clinical care and patient function.

The procedure used in this study can be summarized as a 7-step approach to transforming and synthesizing a raw, multidimensional, hospital patient registry data set into an exploitable database:

1. Write a protocol including a problem statement, research questions or hypotheses, and data extraction methods incorporating inclusion and exclusion criteria.
2. Explore the hospital register’s data catalog (content of administrative, clinical, medical, and drug data; frequency of assessment; types of measurement—health scores, structured observations, free text—as well as the period of data available) in collaboration with the hospital’s clinical data warehouse.
3. Request ethical approval from an ethics committee for the use/reuse of existing patient data.
4. Select the most appropriate data for responding to the research questions/hypotheses.
5. Prepare the data set for further analysis by extracting hospital register data into a CSV (.csv) or Excel (.xls) format, cleaning the data in that format’s file and importing the data set into a statistical package such as R, SPSS, or STATA.
6. Analyze missing data and strategies to address missing values based on best practice.
7. Synthesize the data with regard to the research questions by recoding and clustering.

### Strengths and Limitations

The strengths of our retrospective registry study lie in its huge sample, allowing us to explore the data’s variability and homogeneity in depth. Clustering data risks reducing their variability and the information that can be extracted from them, and some clinical variables showed a significant number of missing values. This fact raises questions about the accuracy and quality of the clinical data assessed, which would require measures of interrater reliability among the health care professionals inputting data into the registry. However, because this was beyond the study’s aims, we did not explore interrater scores of clinical assessments or health care professionals’ scoring of routinely assessed clinical data.

Another limitation to our study was that the sample was restricted to inpatients aged 65 years or older. Because this retrospective, register-based study was part of a larger project [50] focused on medication management among polymedicated, home-dwelling older adults with multiple chronic conditions, we did not have the ethics committee’s approval to extend our extraction of data from the hospital register to all hospitalized adults. Furthermore, our analysis did not consider medicines prescribed before hospital admissions due to a lack of data accuracy and validity.

Finally, and surprisingly, our hospital data set revealed a low mortality rate. Considering the incidence of death in the region, our database showed that it was limited in its representativeness of mortality. Older inpatients presenting with a severe functional decline or at the end of their life probably left the hospital early to die at home or in a nursing home/intermediate care clinic.

### Research Perspectives

Transforming and synthesizing electronic health records is an intermediate stage in the process of subsequently investigating risk profiles and predictive and survival outcomes. Proceeding to these types of analyses requires that each patient has a personal identifier (PID) for computing survival, predictive risk factors, re-admission rates, unplanned institutionalization, and other clinical outcomes explored in cohort and case–control studies. In addition, survival analysis must be performed up to 18 months after discharge—beyond our data analysis cut-off point. Within the framework of a trajectory analysis of health care, all the longitudinal data on 1 patient should be on the same horizontal line in the spreadsheet used for calculations. To do this, each patient must have a unique code allowing data to be linked across multiple hospitalizations. Risk and predictive analyses could be organized using multiple linear logistic regression models (generalized estimating equation [GEE statistics]).

In this study, the data synthesized to date will enable our research to be completed with additional longitudinal survival analyses. The construction of sequences of hospitalizations and rehospitalizations will allow us to better understand the impact of certain events from a longitudinal perspective. The registry data have some limitations because observations are equally spaced in time and all start from the same point, in 2015. However, this study promises to provide valid and robust results, because, despite the sample period, the next hospitalization may in fact be the best measure of treatment impact. For instance, the consequences of treatment decisions taken during one hospitalization (such as medications prescribed or surgical interventions) might only be measurable when the older inpatient needs to be rehospitalized. Yet those unequal periods between hospitalizations may actually prove to be advantageous because they provide a period of effect—that is, a period selected naturally by the evolving health status specific to each older inpatient (eg, inappropriate treatments make inpatients return to hospital at the exact moment their health worsens). A survival analysis would need to be performed to measure the impact of each important intervention (medical act or medication prescription).

### Conclusions

This retrospective registry analysis study delivered a method to transform and synthesize a large, raw data set, which included patients’ health records with sociodemographic, clinical, medical, health status, and medication data. Data were cleaned-up and the most appropriate approach for managing missing values was applied. The multicomponent data synthesis strategy integrated recoding together with empirical and evidence-based statistical clustering methods. Seven clusters were constructed to present the health status of hospitalized older adult inpatients. Medical status, comorbidity, and medication data were recoded to summarize the large data set. Finally, our overall strategy delivered an exploitable, population-based database for the advanced analysis of descriptive, predictive, and survival statistics for older inpatients.

## Appendix

Multimedia Appendix 1 Sociodemographic characteristics, frequencies and hospital lengths of stay of older adult inpatients (N=20,422) for the period 2015–2018.

Multimedia Appendix 2 Distributions of the somatic status of hospitalized older inpatients at hospital discharge (N=20,422).

Multimedia Appendix 3 Distributions of cognitive status data for hospitalized older inpatients (N=20,422).

Multimedia Appendix 4 Distributions of ICD-10 and CHOP data for hospitalized older inpatients (N=20,422).

Multimedia Appendix 5 Distribution of the number of medicines at hospital discharge (N=20,422).

Multimedia Appendix 6 Distributions of prescribed medicines for discharged older adult inpatients based on the first level of the ATC classification system (N=20,422).

Multimedia Appendix 7 Distribution of the number of deteriorated health conditions among the sample of hospitalized older inpatients (N=20,422).

## Abbreviations

ATC: Anatomical Therapeutic Chemical classification system
ICD-10: 10th revision of the International Statistical Classification of Diseases and Related Health Problems.
MRPs: medication-related problems
NA: not available
