# Supplementary material for: Transforming a Patient Registry Into a Customized Data Set for the Advanced Statistical Analysis of Health Risk Factors and for Medication-Related Hospitalization Research: Retrospective Hospital Patient Registry Study
Source: JMIR Med Inform. 2021 May 11;9(5):e24205. doi: 10.2196/24205 (PMC8150425; doi:10.2196/24205)
Supplement: Multimedia Appendix 1 [file medinform_v9i5e24205_app1.docx]

**Multimedia Appendix**

This is a Multimedia Appendix to a full manuscript published in the J Med Internet Res. For full copyright and citation information see http://dx.doi.org/10.2196/24205

Supplementary Table 1. Sociodemographic characteristics, frequencies and hospital lengths of stay of older adult inpatients (N = 20,422) for the period 2015–2018.

| **Characteristics** | **Distribution – n (%) or mean (SD)** |
| --- | --- |
| **Sex**  Male (%)  Female (%) | 10,720 (52.5)  9,702 (47.5) |
| **Age (years)**  Average (SD)  Median  Min.–Max. | 78.9 (7.7)  79.0  65–107 |
| **Hospital length of stay (days)**  Average (SD)  Median  Min.–Max. days of hospitalisation | 10.5 (9.6)  8.0  1–204 |
| **Place of discharge**  Home  Long-term care facilities  Rehabilitation settings  Died during hospitalisation  Unknown/other  Missing values | 13,802 (68.2)  632 (3.1)  5,600 (27.5)  131 (0.6)  86 (0.4)  171 (0.8) |
| **Rehospitalisation**  No rehospitalisation  1–2  3–4  5–6  7–8  9–10  11–12  ≥ 13  Average (SD)  Median  Min. –Max. | 7,598 (37.2)  8,146 (39.9)  2,831 (13.9)  1,121 (5.5)  406 (2.0)  184 (0.9)  73 (0.4)   1. (0.3)   2.6 (2.1)  2  0–17 |
