# Supplementary material for: Transforming a Patient Registry Into a Customized Data Set for the Advanced Statistical Analysis of Health Risk Factors and for Medication-Related Hospitalization Research: Retrospective Hospital Patient Registry Study
Source: JMIR Med Inform. 2021 May 11;9(5):e24205. doi: 10.2196/24205 (PMC8150425; doi:10.2196/24205)
Supplement: Multimedia Appendix 2 [file medinform_v9i5e24205_app2.docx]

**Multimedia Appendix**

This is a Multimedia Appendix to a full manuscript published in the J Med Internet Res. For full copyright and citation information see http://dx.doi.org/10.2196/24205

Supplementary Table 2. Distributions of the somatic status of hospitalised older inpatients at hospital discharge (N = 20,422).

| **Variables** | **Distribution** |
| --- | --- |
| **Mobility–Moving**  Complete capacity  Mild incapacity  Strong incapacity  Total incapacity  Not available | 11,403 (55.8)  5,997 (29.4)  2,403 (11.8)  615 (3.0)  4 (< 0.01) |
| **Position change**  Complete capacity  Mild incapacity  Strong incapacity  Total incapacity  Not available | 14,358 (70.3)  4,118 (20.2)  1,636 (8.0)  307 (1.5)  1 (< 0.01) |
| **Altered gait**  No  Yes  Not evaluable  Not available | 11,626 (56.9)  8,323 (40.8)  471 (2.3)  2 (< 0.01) |
| **Falls in last year**  No falls  1–3  4–6  7–9 | 6,744 (33.1)  10,376 (51.0)  3,042 (15.0)  185 (0.9) |
| **Exhaustion**  Mental and physical strength maintained  Some activities possible  Some activities possible with recuperation  No activities possible  Not evaluable  Not available | 9,587 (46.9)  7,700 (37.7)  2,792 (13.7)  320 (1.6)  20 (0.1)  3 (< 0.01) |
| **Upper-body care**  Complete capacity  Mild incapacity  Severe incapacity (help)  Total incapacity  Not available | 13,904 (68.1)  4,321 (21.2)  1,508 (7.4)  684 (3.3)  5 (< 0.01) |
| **Lower-body care**  Complete capacity  Mild incapacity  Severe incapacity  Total incapacity  Not available | 10,965 (53.7)  4,506 (22.1)  2,599 (12.7)  2,347 (11.5)  5 (< 0.01) |
| **Upper-body dressing and undressing**  Complete capacity  Mild incapacity  Severe incapacity  Total incapacity  Not available | 14,290 (70.0)  3,598 (17.6)  1,533 (7.5)  995 (4.9)  6 (< 0.01) |
| **Lower-body dressing and undressing**  Complete capacity  Mild incapacity  Severe incapacity  Total incapacity  Not available | 11,461 (56.1)  4,034 (19.8)  2,542 (12.4)  2,379 (11.6)  6 (< 0.01) |
| **Eating-related movements**  Complete capacity  Mild incapacity  Severe incapacity  Total incapacity  Not available | 17,792 (87.2)  1,811 (8.9)  451 (2.2)  360 (1.8)  8 (< 0.01) |
| **Drinking-related movements**  Complete capacity  Mild incapacity  Severe incapacity  Total incapacity  Not available | 18,813 (92.1)  1,026 (5.0)  337 (1.7)  241 (1.2)  5 (< 0.01) |
| **Micturition-related movements**  Complete capacity  Mild incapacity  Severe incapacity  Total incapacity  Not available | 15,452 (75.7)  2,497 (12.2)  933 (4.6)  1,525 (7.5)  15 (0.1) |
| **Defecation-related movements**  Complete capacity  Mild incapacity  Severe incapacity  Total incapacity  Not available | 16,099 (78.8)  2,268 (11.1)  1,151 (5.6)  891 (4.4)  13 (0.1) |
| **Hearing**  No deficiency  Hearing deficiency  Deaf  Not evaluable  Not available | 17,618 (86.3)  2,726 (13.3)  38 (0.2)  19 (0.1)  21 (0.1) |
| **Vision**  No visual deficiency  Visual deficiency  Blind  Not evaluable  Not available | 18,204 (89.1)  2,129 (10.4)  33 (0.2)  35 (0.2)  21 (0.1) |
| **Verbal expression**  Complete capacity  Mild incapacity  Severe incapacity  Total incapacity  Not available | 19,096 (93.5)  1,180 (5.8)  -  126 (0.6)  20 (0.1) |
| **Pain intensity**  No pain  Probably no pain  Mild pain (VAS^a^ 1–3)  Some indication of pain (self-evaluation)  Moderate-intensity pain (VAS^a^ 4–6)  High-intensity pain (VAS^a^ 7–10)  Not available | 1,790 (8.8)  5,278 (25.8)  228 (1.1)  13,040 (63.9)  19 (0.1)  26 (0.1)  41 (0.2) |
| **Chronic pain**  No  Yes  Not evaluable  Not available | 18,253 (89.4)  2,074 (10.2)  46 (0.2)  49 (0.2) |

^a^ VAS = Visual Analog Scale for Pain
