# Supplementary material for: Transforming a Patient Registry Into a Customized Data Set for the Advanced Statistical Analysis of Health Risk Factors and for Medication-Related Hospitalization Research: Retrospective Hospital Patient Registry Study
Source: JMIR Med Inform. 2021 May 11;9(5):e24205. doi: 10.2196/24205 (PMC8150425; doi:10.2196/24205)
Supplement: Multimedia Appendix 3 [file medinform_v9i5e24205_app3.docx]

**Multimedia Appendix**

This is a Multimedia Appendix to a full manuscript published in the J Med Internet Res. For full copyright and citation information see http://dx.doi.org/10.2196/24205

Supplementary Table 3. Distributions of cognitive status data for hospitalised older inpatients (N = 20,422).

| **Variables** | **Distribution (%)** |
| --- | --- |
| **Perception–alertness**  Alert  Drowsy  Stupor  Comatose  Not available | 20,050 (98.2)  308 (1.5)  26 (0.1)  17 (0.1)  21 (0.1) |
| **Orientation**  Possesses full faculties  Possesses three faculties (recognise time, place, relatives )  Possesses one or two faculties  Possesses no faculties  Not evaluable  Not available | 16,925 (82.9)  1,905 (9.3)  1,014 (5.0)  420 (2.1)  138 (0.7)  20 (0.1) |
| **Attention**  No alteration  Permanently restricted  Not evaluable  Not available | 18,728 (91.7)  1,653 (8.1)  21 (0.1)  20 (0.1) |
| **Skills for daily life (decision making process)**  No restriction  Mild restriction  Severe restriction  No evaluable  Not available | 15,323 (75.0)  3,008 (14.7)  2,040 (10.0)  31 (0.2)  20 (0.1) |
| **Ability to learn**  Fully capable  Mild restriction  Severe restriction  Totally incapable  Not available | 14,826 (72.6)  3,452 (16.9)  1,681 (8.2)  443 (2.2)  20 (0.1) |
