# Supplementary material for: Transforming a Patient Registry Into a Customized Data Set for the Advanced Statistical Analysis of Health Risk Factors and for Medication-Related Hospitalization Research: Retrospective Hospital Patient Registry Study
Source: JMIR Med Inform. 2021 May 11;9(5):e24205. doi: 10.2196/24205 (PMC8150425; doi:10.2196/24205)
Supplement: Multimedia Appendix 4 [file medinform_v9i5e24205_app4.docx]

**Multimedia Appendix**

This is a Multimedia Appendix to a full manuscript published in the J Med Internet Res. For full copyright and citation information see http://dx.doi.org/10.2196/24205

Supplementary Table 4. Distributions of ICD-10 and CHOP data for hospitalised older inpatients (N = 20,422).

| **Variables** | **Distribution (%)** |
| --- | --- |
| **Medical diagnosis ICD-10**  ***Principal diagnosis***  Diagnosed with organic disorders  Diagnosed with mental disorders  Diagnosed with oncological diseases  Other  Not available  ***Distribution of principal and secondary diagnoses***  1 ICD-10  2 ICD-10  3 ICD-10  4 ICD-10  5 ICD-10  **Swiss *surgical classification system* (CHOP)**  No surgery  Sensory system surgical intervention  Organic system surgical intervention  Other  ***Distribution of principal and secondary CHOP***  No surgery  1 CHOP  2 CHOP  3 CHOP  4 CHOP  ≥ 5 CHOP | 10,666 (52.2)  2,041 (10.0)  221 (1.1)  7,490 (36.7)  4 (< 0.01)  327 (1.6)  677 (3.3)  1,150 (5.6)  1,461 (7.2)  16,807 (82.3)  6,275 (30.7)  526 (2.6)  5,086 (24.9)  8,535 (41.8)  6,274 (30.7)  4,057 (19.9)  3,285 (16.1)  3,222 (15.8)  2,115 (10.4)  1,469 (7.2) |
