# Supplementary material for: Transforming a Patient Registry Into a Customized Data Set for the Advanced Statistical Analysis of Health Risk Factors and for Medication-Related Hospitalization Research: Retrospective Hospital Patient Registry Study
Source: JMIR Med Inform. 2021 May 11;9(5):e24205. doi: 10.2196/24205 (PMC8150425; doi:10.2196/24205)
Supplement: Multimedia Appendix 5 [file medinform_v9i5e24205_app5.docx]

**Multimedia Appendix**

This is a Multimedia Appendix to a full manuscript published in the J Med Internet Res. For full copyright and citation information see http://dx.doi.org/10.2196/24205

Supplementary Table 5. Distribution of the number of medicines at hospital discharge (N = 20,422).

| **Number of medicines** | **Frequency (%)** |
| --- | --- |
| 5 medicines | 2,094 (10.3) |
| 6 medicines | 2,233 (10.9) |
| 7 medicines | 2,353 (11.5) |
| 8 medicines | 2,281 (11.2) |
| 9 medicines | 2,169 (10.6) |
| 10 medicines | 1,951 (9.6) |
| 11 medicines | 1,706 (8.4) |
| 12 medicines | 1,373 (6.7) |
| 13 medicines | 1,135 (5.6) |
| 14 medicines | 923 (4.5) |
| 15 medicines | 663 (3.2) |
| 16 medicines | 501 (2.5) |
| 17 medicines | 306 (1.5) |
| 18 medicines | 248 (1.2) |
| 19 medicines | 153 (0.7) |
| 20 medicines | 105 (0.5) |
| 21–40 medicines | 228 (1.1) |
