# Supplementary material for: Transforming a Patient Registry Into a Customized Data Set for the Advanced Statistical Analysis of Health Risk Factors and for Medication-Related Hospitalization Research: Retrospective Hospital Patient Registry Study
Source: JMIR Med Inform. 2021 May 11;9(5):e24205. doi: 10.2196/24205 (PMC8150425; doi:10.2196/24205)
Supplement: Multimedia Appendix 6 [file medinform_v9i5e24205_app6.docx]

**Multimedia Appendix**

This is a Multimedia Appendix to a full manuscript published in the J Med Internet Res. For full copyright and citation information see http://dx.doi.org/10.2196/24205

Supplementary Table 6. Distributions of prescribed medicines for discharged older adult inpatients based on the first level of the ATC classification system (N = 20,422).

| **Types of medicines prescribed (N = 2,370) based on the first level of the Anatomical Therapeutic Chemical classification system** | **Frequency (%)** |
| --- | --- |
| Alimentary tract and metabolism  Blood and blood-forming organs  Cardiovascular system  Dermatologicals  Genito-urinary system and sex hormones  Systemic hormonal preparations, excluding sex hormones and insulins  Anti-infectives for systemic use  Antineoplastic and immunomodulating agents  Musculoskeletal system  Nervous system  Antiparasitic products, insecticides and repellents  Respiratory system  Sensory organs  Various | 376 (15.8)  117 (4.9)  574 (24.2)  86 (3.6)  83 (3.5)  65 (2.7)  148 (6.2)  75 (3.2)  104 (4.4)  514 (21.7)  8 (0.4)  122 (5.2)  87 (3.7)  11 (0.5) |
