# Supplementary material for: Transforming a Patient Registry Into a Customized Data Set for the Advanced Statistical Analysis of Health Risk Factors and for Medication-Related Hospitalization Research: Retrospective Hospital Patient Registry Study
Source: JMIR Med Inform. 2021 May 11;9(5):e24205. doi: 10.2196/24205 (PMC8150425; doi:10.2196/24205)
Supplement: Multimedia Appendix 7 [file medinform_v9i5e24205_app7.docx]

**Multimedia Appendix**

This is a Multimedia Appendix to a full manuscript published in the J Med Internet Res. For full copyright and citation information see http://dx.doi.org/10.2196/24205

Supplementary Table 7. Distribution of the number of deteriorated health conditions among the sample of hospitalised older inpatients (N = 20,422).

| **Number of deteriorated health conditions** | **Frequency (%)** |
| --- | --- |
| No deteriorated health conditions | 11,409 (55.9) |
| 1 deteriorated health condition | 6,947 (34.0) |
| 2 deteriorated health conditions | 1,394 (6.8) |
| 3 deteriorated health conditions | 393 (1.9) |
| 4 deteriorated health conditions | 160 (0.8) |
| 5 deteriorated health conditions | 75 (0.4) |
| 6 deteriorated health conditions | 19 (< 0.0) |
